# Individual and joint associations between sleep duration and physical activity with cognitive function: A longitudinal analysis among middle‐aged and older adults in China

**DOI:** 10.1002/alz.14212

**Published:** 2024-12-18

**Authors:** Hongwei Liu, Yan Shi, Min Yu, Xiaolei Guo, Ye Ruan, Fei Qin, Rongfei Zhou, Jingyuan Feng, Zihan Hu, Fei Wu, Qingqing Jia, Yanlu Yin, Yanfei Guo, Fan Wu

**Affiliations:** ^1^ School of Public Health Fudan University Shanghai China; ^2^ Division of Chronic Non‐Communicable Disease and Injury Shanghai Municipal Center for Disease Control and Prevention Shanghai China; ^3^ Zhejiang Provincial Center for Disease Control and Prevention Hangzhou China; ^4^ The Department for Chronic and Non‐Communicable Disease Control and Prevention Shandong Center for Disease Control and Prevention Jinan China; ^5^ School of Public Health and Community Medicine, Institution of Medicine, Sahlgrenska Academy University of Gothenburg Gothenburg Sweden

**Keywords:** cognitive function, middle‐aged and older adults, nocturnal sleep duration, physical activity

## Abstract

**INTRODUCTION:**

Studies using cross‐sectional data or with a short follow‐up period fail to distinguish whether the associations between sleep duration and physical activity with cognitive function result from reverse causation.

**METHODS:**

The longitudinal study examined the individual and joint associations, with specific temporality, between sleep duration and physical activity with cognitive function, using time‐lagged linear mixed models and generalized additive mixed models.

**RESULTS:**

A total of 14,694 participants aged ≥ 50 years were included, with an average lagged time of 4.5 (standard deviation 1.3) years. Long sleep duration was independently associated with cognitive decline, while short sleep duration and physical activity were not. The analysis of joint effects showed that increased physical activity slowed the rate of cognitive decline among participants reporting long sleep duration, consistent with the results of the stratified analyses.

**DISCUSSION:**

Interventions on improving sleep should consider concurrent physical activity to maximize benefits for slowing cognitive decline.

**Highlights:**

- Long sleep duration was independently associated with worse cognitive function, while short sleep duration was not.
- Elevated levels of physical activity were not independently associated with better cognitive function.
- Increased physical activity appeared to mitigate the negative impact of long sleep duration on cognitive function.

## 1 BACKGROUND

As a leading cause of cognitive dysfunction and disability in older adults, dementia has posed a significant public health burden with the global population aging.^1^ The World Alzheimer Report 2018 indicates that the number of people with dementia is ≈ 50 million worldwide, and is projected to reach 152 million by 2050.^2^ In China, > 5% of older adults (aged ≥ 60) are reported to have dementia, accounting for 25% of patients with dementia in the world.^1^, ^3^ Dementia is a progressive neurodegenerative disorder that has a long preclinical period characterized by cognitive decline.^4^ In the absence of effective treatment, identifying modifiable risk factors is crucial to improve cognitive function and delay the onset of dementia.^5^ Although considerable evidence has found positive effects of improved sleep and increased physical activity on cognitive function, emerging recent studies report inconsistent results.

In the preclinical period of dementia, individuals commonly experience behavioral changes such as alterations in sleep patterns and physical activity.^6^ Thus, studies using cross‐sectional data or with short follow‐up times cannot effectively distinguish whether the associations between sleep duration and physical activity with cognitive function are due to preclinical changes of dementia (i.e., reverse causation).^7^, ^8^ The protective effect of increased physical activity and maintaining medium sleep duration on cognitive function is more reported in cross‐sectional studies or studies with short follow‐up times.^9^, ^10^, ^11^ The results from studies with long follow‐up times are quite inconsistent.^7^, ^8^, ^12^ For example, a study among 2968 Japanese community‐dwelling older adults revealed that regular physical activity in midlife was associated with lower odds of late‐life mild cognitive impairment (MCI).^13^ Another pooled study, combining two nationally representative aging cohorts, found that both insufficient and excessive sleep duration were associated with faster cognitive decline.^14^ However, a recent study using data from the English Longitudinal Study of Ageing found that higher physical activity and medium sleep duration were associated with better cognitive function at baseline, while no significant differences in 10‐year cognitive decline across different levels of physical activity or between medium and long sleep durations.^8^

Sleep and physical activity are vital health behaviors that influence each other.^15^ Numerous studies have demonstrated that increased physical activity can improve sleep patterns^16^ and attenuate the adverse effects of poor sleep on various health outcomes, such as metabolic diseases, hypertension, and all‐cause mortality.^17^, ^18^ The modification effect of physical activity on sleep may derive from the fact that higher levels of physical activity can mitigate oxidative stress, inflammation, and vascular endothelium damage induced by poor sleep, which play a crucial role in the onset and progression of dementia.^19^ However, the large‐scale population‐based evidence, especially with long follow‐up periods, regarding the association between the combination of physical activity and sleep duration with cognitive function is limited.

The aim of this study was to examine the individual and joint longitudinal associations between nocturnal sleep duration and physical activity with cognitive function, using data from a nationally representative population‐based cohort. In addition, we also examined the potential modifying effect of physical activity on the association between sleep duration and cognitive function.

## 2 METHODS

### 2.1 Study design and participants

Data from three waves of the World Health Organization (WHO)’s Study on Global Ageing and Adult Health (SAGE) in China were analyzed. SAGE was a longitudinal cohort study with nationally representative samples of persons aged ≥ 50 years, conducted in six low‐ and middle‐income countries, including China, Ghana, India, Mexico, Russia, and South Africa. A stratified multistage random cluster sampling design was used to obtain the nationally representative sample of individuals ≥ 50 years, along with a smaller sample of individuals aged 18 to 49 years for comparisons. More details of SAGE have been published elsewhere.^20^

In China, the SAGE was conducted in 32 urban and 32 rural areas across seven provinces (Jilin, Shandong, Shaanxi, Hubei, Zhejiang, Guangdong, and Yunnan) and one municipality (Shanghai). Data were collected through face‐to‐face household interviews using questionnaires covering demographic, socioeconomic, social, behavioral, and health information. Before the survey commenced, participants’ cognitive performance was assessed with a set of questions. In the event of cognitive limitations, the proxy of the participant completed the proxy questionnaire; otherwise, the participant completed the individual questionnaire.^20^ To reduce the impact of the preclinical phase of dementia on physical activity and sleep, the study only included the participants completing the individual questionnaire (i.e., without cognitive limitations).

A total of 22,642 participants, completing the individual questionnaire, were recruited during the baseline survey (2007–2010, wave 1), with follow‐ups in 2014 to 2015 (19,167 participants, wave 2) and 2017 to 2018 (15,006 participants, wave 3). New participants were added in each follow‐up to increase the sample size. Eventually, a total of 14,694 participants and 22,617 observations were included in the main analysis. Out of 14,694 participants included, 6771 participants completed two cognitive measurements, and 7923 participants completed three. The detailed flowchart of the participant selection and exclusion process is presented in Figure S1 in supporting information.

The study was approved by the ethics committee of the Chinese Centre for Disease Control and Prevention (approval number: 200601) for the baseline investigation and the Shanghai Municipal Center for Disease Control and Prevention (approval numbers: 2014‐8, 2018–1) for the follow‐up investigations. Informed consent was obtained from all participants before the interview.

### 2.2 Sleep duration and physical activity

Participants reported sleep duration for the two nights before the survey in hours and minutes. The values from the two nights were averaged to create a composite measure of sleep length.^11^ According to previous studies, sleep duration was categorized into three groups: short sleep duration (< 6 hours per night), medium sleep duration (6–8 hours per night), and long sleep duration (> 8 hours per night).^21^

Physical activity in a typical week was assessed using the Global Physical Activity Questionnaire (GPAQ).^22^ Participants reported their weekly frequency and duration of physical activities in five domains: vigorous activity at work, moderate activity at work, travel to and from places, vigorous recreational activities, and moderate recreational activities. Metabolic equivalents (METs) were used to indicate the intensity of physical activities in the GPAQ data analysis. In GPAQ analysis, four METs are designated for moderate activities and transportation, while eight METs are attributed to vigorous activities. The cumulative METs minutes (METs‐min) per week for moderate and vigorous intensity activities and transportation were subsequently computed and combined. Participants were categorized into three groups based on WHO recommendations for total physical activity: high physical activity (> 300 minutes of moderate‐intensity activity, > 150 minutes of vigorous‐intensity activity, or an equivalent combination totaling at least 1200 METs‐min per week), moderate physical activity (150–300 minutes of moderate‐intensity activity, 75–150 minutes of vigorous‐intensity activity, or an equivalent combination totaling 600–1200 METs‐min per week), and low physical activity (not meeting these recommendations).^23^

**RESEARCH IN CONTEXT**

1. **Systematic review**: The preclinical period of dementia commonly results in alterations in sleep patterns and physical activity. Thus, studies using cross‐sectional data or with short follow‐up periods cannot effectively distinguish whether the associations between sleep duration and physical activity with cognitive function are due to preclinical changes of dementia (i.e., reverse causation).
2. **Interpretation**: The study suggested that long sleep duration was independently associated with cognitive function at follow‐up, while short sleep duration and physical activity were not. This was further verified in the curvilinear relationships between sleep duration and physical activity with cognitive function. Our results also found that increased physical activity may attenuate the detrimental effects of long sleep duration on cognitive function.
3. **Future directions**: Future studies are warranted to examine: (a) the effects of short sleep duration on cognitive function across stratum of age and determine optimal sleep duration for different strata of age; and (b) the effects of different types and intensity of physical activity on cognitive function.

### 2.3 Cognitive function

Five standardized cognitive tests were administered to participants at both baseline and follow‐up. These tests covered different domains of cognitive function, including verbal learning and recall (immediate and delayed verbal recall), attention and working memory (forward and backward digit span), and executive function (verbal fluency). A summary variable representing overall cognitive performance was created based on the results of these tests for each participant.^24^

In the immediate verbal recall test, the interviewer read a list of words, then the respondents were given 1 minute to repeat as many words as possible. This test was repeated three times. Upon completing the third trial, other cognitive tests were conducted. A delayed verbal recall test followed, in which respondents recalled words again without the interviewer repeating the list. Each correctly recalled word was recorded as one point. For the forward and backward digit span test, the respondents were asked to repeat a string of numbers forward and backward, respectively, with the number of the longest error‐free series repeated recorded as the total score. To assess verbal fluency, the respondents were given 1 minute to name as many animals as possible, with one point scored for each correct answer.

Composite *z* scores were calculated to represent overall cognitive performance, allowing for comparison between participants.^10^, ^11^ Each test score was standardized based on the mean and standard deviation of the baseline test scores, and the composite *z* score was calculated by averaging the *z* scores from the five tests.^14^ Finally, composite *z* scores were converted to a 0 to 100 scale, with higher scores indicating higher cognitive performance for each participant.

### 2.4 Covariates

Sociodemographic variables included age (years), sex (male or female), education level (no formal education, less than high school, or high school and above), marital status (married or unmarried), employment status (working, retired, or never worked), and socioeconomic status (low or high). The monthly household consumption spending per capita was used as a proxy for socioeconomic status.^25^

Health‐related behaviors covered smoking status (current smokers, former smokers, or never smokers), alcohol consumption (lifetime abstainers, not‐heavy drinkers, or heavy drinkers), sedentary behavior (low, moderate, or high), social cohesion, and daily vegetable and fruit intake (insufficient or sufficient). A social cohesion index was established on a scale of 9 to 45, with higher scores denoting greater social cohesion.^26^

Health conditions included rapid gait speed (m/s), sleep quality, body mass index (BMI; underweight, normal weight, or overweight and obesity), depression (yes or no), stroke (yes or no), and chronic disease comorbidity status (no chronic condition, 1–2 types of chronic conditions, or ≥ 3 types of chronic conditions). Rapid gait speed was measured as a proxy for physical function. The total score of sleep quality ranged from 1 to 5, with higher scores denoting poor sleep quality. The total number of seven chronic disease conditions (hypertension, arthritis, angina, diabetes, chronic lung disease, asthma, and cataracts) was aggregated to represent chronic disease comorbidity status.

The practice effect, indicating the round of cognitive testing for each participant, reflected the tendency for participants’ cognitive scores to improve with successive rounds of testing due to familiarity with the testing protocol.^27^ The detailed definitions of covariates were provided in Table S1 in supporting information.

### 2.5 Statistical analysis

Baseline characteristics of the participants were presented as the mean (standard deviation [SD]) for continuous variables and as frequency (percentage) for categorical variables across the categories of sleep duration (short, medium, and long). Multiple imputation using the chained‐equations method was conducted to impute missing covariate data and generate five imputed datasets for the subsequent analyses.

Time‐lagged models were used to examine the lagged effects of sleep duration and physical activity at each wave on cognitive function in subsequent waves while controlling for the autoregression of cognitive function (i.e., the effect of cognitive function at each wave on cognitive function in subsequent waves). Using exposure variables from previous waves to predict future outcome variables in models is a valuable approach for examining the longitudinal association between variables, demonstrating temporality.^28^

Time‐lagged linear mixed models (LMMs) were used to identify the longitudinal individual associations between sleep duration and physical activity with cognitive function. LMMs could take the interdependency of repeated measures within persons into account, suitable for analyzing repeated measurement data or longitudinal panel data.^29^ Three models were conducted. Model 1 included sleep duration and physical activity simultaneously and adjusted for age, sex, education level, practice effect, rapid gait speed, sedentary behavior, sleep quality, composite cognitive score at the concurrent wave, and the interval between the concurrent and next wave. Model 2 further adjusted for marital status, employment status, socioeconomic status, smoking status, alcohol consumption, social cohesion, and daily vegetable and fruit intake. Based on Model 2, Model 3 adjusted for BMI, depression, stroke, and chronic disease comorbidity status.

Time‐lagged generalized additive mixed models were used to further explore longitudinal non‐linear associations between sleep duration and physical activity with cognitive function with adjustment for full covariates. In the model, sleep duration and physical activity were treated as continuous variables. Cubic regression splines were fitted to the associations between target variables with the number of degrees of freedom chosen based on generalized cross‐validation (GCV).

A stratified analysis by levels of physical activity was conducted to explore the potential modifying effect of physical activity on the longitudinal association between sleep duration and cognitive function. The statistical differences in associations between the groups were tested using the likelihood ratio test by comparing the inclusion and exclusion of the cross‐product interaction term of physical activity and sleep duration in the models.^30^

To further quantify the joint association of sleep duration and physical activity with cognitive function, we developed additional time‐lagged LMMs, with a joint variable that combined sleep duration and physical activity.^17^ The joint variable was classified into nine groups according to categories of sleep duration (short, medium, and long) and physical activity (low, moderate, and high) with the group that slept 6 to 8 hours per night and engaged in high physical activity as the reference group. The settings of models were consistent with the analyses of individual associations.

We conducted a series of sensitivity analyses to examine the robustness of our findings and reduce the possibility of reverse causation. First, to further demonstrate the longitudinal associations between sleep duration and physical activity with cognitive function, we examined the individual associations between sleep duration and physical activity at wave 1 with cognitive function at wave 3 with an average lagged time of 8.93 years. Second, to reduce the potential impact of possible reverse causation, we repeated the analyses in which participants with MCI in at least two waves were identified and excluded.^8^ Third, we restricted the analyses to participants with complete covariate data for comparison to the results through multiple imputation. Fourth, we repeated the analyses with the categorization of 7 to 8 hours per night as medium sleep duration and the reference group.

All models used restricted maximum likelihood estimation and included the intercept as a random effect. The data analyses were conducted using R (version 4.2.3) with the “nlme” package for linear mixed effects models, the “gamm4” package for generalized additive mixed models, the “modelr” package for GCV, and the “mice” package for multiple imputations. Statistical significance was set at a two‐tailed *p* < 0.05.

## 3 RESULTS

A total of 14,694 participants and 22,617 observations were included in the main analysis, with an average lagged time of 4.5 (SD 1.3) years between the concurrent wave and the next wave. Table 1 shows the baseline characteristics of participants by sleep duration. At baseline, the mean age was 62.1 (SD 9.5) years and 45.6% of the participants were male. The missing rate of covariates is presented in Table S2 in supporting information; the baseline characteristics of participants across subgroups combining sleep duration and physical activity in Table S3 in supporting information; the distributions of sleep duration and physical activity in Figures S2 and S3 in supporting information; the correlations among sleep duration, physical activity, and sedentary behavior in Table S4 in supporting information; and the multicollinearity among variables in the mixed models in Table S5 in supporting information.

**TABLE 1:** Baseline characteristics of the participants by sleep duration.

		Sleep duration		
	Total (*n* = 14,694)	Short (*n* = 2207)	Medium (*n* = 7950)	Long (*n* = 4537)
Physical activity, *n* (%)				
Low	5686 (38.7)	846 (38.3)	3000 (37.7)	1840 (40.6)
Moderate	1890 (12.9)	287 (13.0)	1090 (13.7)	513 (11.3)
High	7118 (48.4)	1074 (48.7)	3860 (48.6)	2184 (48.1)
Age, years, mean (SD)	62.1 (8.5)	63.4 (8.7)	61.3 (8.2)	62.7 (8.9)
Sex, *n* (%)				
Female	7980 (54.3)	1220 (55.3)	4312 (54.2)	2448 (54.0)
Male	6714 (45.7)	987 (44.7)	3638 (45.8)	2089 (46.0)
Education level, *n* (%)				
No formal education	1856 (12.6)	292 (13.2)	774 (9.7)	790 (17.4)
Less than high school	9334 (63.5)	1408 (63.8)	4963 (62.4)	2963 (65.3)
High school and above	3504 (23.8)	507 (23.0)	2213 (27.8)	784 (17.3)
Marital status, *n* (%)				
Unmarried	1848 (12.6)	369 (16.7)	890 (11.2)	589 (13.0)
Married	12,846 (87.4)	1838 (83.3)	7060 (88.8)	3948 (87.0)
Employment status, *n* (%)				
Never worked	1623 (11.1)	273 (12.4)	794 (10.0)	556 (12.3)
Retired	7134 (48.6)	1158 (52.5)	4009 (50.5)	1967 (43.4)
Working	5927 (40.4)	776 (35.2)	3143 (39.6)	2008 (44.3)
Socioeconomic status, *n* (%)				
Low	7183 (49.2)	1078 (49.2)	3539 (44.8)	2566 (56.9)
High	7418 (50.8)	1111 (50.8)	4366 (55.2)	1941 (43.1)
Rapid gait speed, m/s, mean (SD)	1.4 (0.4)	1.4 (0.4)	1.4 (0.4)	1.4 (0.4)
Sedentary behavior, *n* (%)				
Low	4496 (30.7)	639 (29.0)	2522 (31.8)	1335 (29.5)
Moderate	5558 (37.9)	757 (34.4)	2925 (36.9)	1876 (41.5)
High	4592 (31.4)	804 (36.5)	2481 (31.3)	1307 (28.9)
Sleep quality, mean (SD)	2.4 (0.7)	3.0 (0.9)	2.3 (0.7)	2.2 (0.7)
Smoking status, *n* (%)				
Never smokers	9645 (68.5)	1448 (67.9)	5250 (69.6)	2947 (67.1)
Former smokers	675 (4.8)	125 (5.9)	321 (4.3)	229 (5.2)
Current smokers	3752 (26.7)	559 (26.2)	1975 (26.2)	1218 (27.7)
Alcohol consumption, *n* (%)				
Lifetime abstainers	10,024 (71.3)	1488 (69.6)	5421 (72.0)	3115 (70.9)
Non‐heavy drinkers	3301 (23.5)	528 (24.7)	1781 (23.6)	992 (22.6)
Heavy drinkers	741 (5.3)	123 (5.8)	331 (4.4)	287 (6.5)
Social cohesion index, mean (SD)	14.8 (3.7)	14.8 (4.0)	14.8 (3.7)	14.6 (3.5)
Daily vegetable and fruit intake, *n* (%)				
Insufficient	2916 (20.3)	438 (20.3)	1520 (19.6)	958 (21.6)
Sufficient	11,436 (79.7)	1721 (79.7)	6239 (80.4)	3476 (78.4)
BMI, kg/m^2^, *n* (%)				
Underweight (< 18.5)	445 (3.2)	1035 (48.1)	3539 (46.5)	2039 (47.1)
Normal (18.5–24)	6613 (46.9)	99 (4.6)	196 (2.6)	150 (3.5)
Overweight and obesity (≥ 24)	7033 (49.9)	1020 (47.4)	3869 (50.9)	2144 (49.5)
Depression, *n* (%)				
No	14,532 (99.0)	2157 (97.9)	7880 (99.2)	4495 (99.2)
Yes	141 (1.0)	46 (2.1)	61 (0.8)	34 (0.8)
Stroke, *n* (%)				
No	14,193 (96.7)	2109 (95.6)	7700 (97.0)	4384 (96.7)
Yes	482 (3.3)	96 (4.4)	238 (3.0)	148 (3.3)
Chronic disease comorbidity status, *n* (%)				
No	4063 (27.7)	527 (23.9)	2368 (29.8)	1168 (25.7)
1–2 types	9193 (62.6)	1324 (60.0)	4897 (61.6)	2972 (65.5)
≥ 3 types	1438 (9.8)	356 (16.1)	685 (8.6)	397 (8.8)
Baseline composite cognitive score, mean (SD)	61.8 (10.2)	60.8 (10.8)	62.9 (10.0)	60.6 (10.1)

Abbreviations: BMI, body mass index; SD, standard deviation.

**FIGURE 1:**
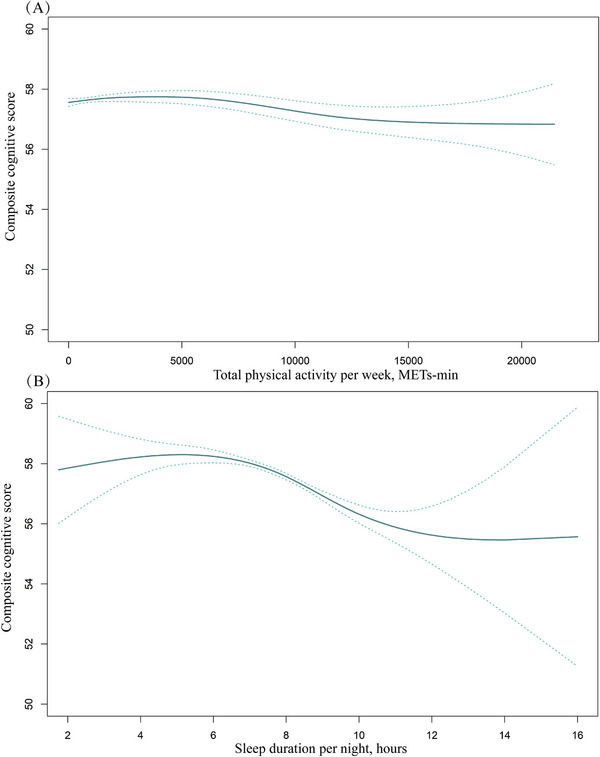
The adjusted dose–response associations between sleep duration and physical activity with cognitive function. (A) displayed the adjusted dose–response associations between physical activity with cognitive function. (B) represented the adjusted dose–response associations between sleep duration with cognitive function. Adjustment for full covariates included age, sex, education level, practice effect, rapid gait speed, sedentary behavior, sleep quality, composite cognitive score at concurrent wave, the interval between concurrent and next wave, marital status, employment status, socioeconomic status, smoking status, alcohol consumption, social cohesion, daily vegetable and fruit intake, BMI, depression, stroke, and chronic disease comorbidity status. The number of degrees of freedom was 5 for sleep duration and 8 for physical activity in models based on GCV (Table S7 in supporting information). BMI, body mass index; GCV, generalized cross‐validation; METs‐min, metabolic equivalent minutes.

### 3.1 Individual associations of sleep duration and physical activity with cognitive function

Table 2 presents the individual associations of sleep duration and physical activity with cognitive function. The results were mostly similar across the three models, showing slight attenuation with covariate adjustments.

**TABLE 2:** The individual associations of sleep duration and physical activity with cognitive function.

	Model 1^a^		Model 2^b^		Model 3^c^	
	*β*	*P*	*β*	*P*	*β*	*P*
Physical activity						
Low	Ref		Ref		Ref	
Moderate	0.40	0.048	0.34	0.087	0.34	0.090
High	−0.11	0.440	0.21	0.126	0.21	0.129
Sleep duration						
Short	0.25	0.209	0.27	0.164	0.30	0.118
Medium	Ref		Ref		Ref	
Long	−1.43	<0.001	−1.17	<0.001	−1.13	<0.001

^a^ Model 1 adjusted for age, sex, education level, practice effect, rapid gait speed, sedentary behavior, sleep quality, composite cognitive score at the concurrent wave, and the interval between the concurrent and next wave.

^b^ Model 2 additionally adjusted for marital status, employment status, socioeconomic status, smoking status, alcohol consumption, social cohesion, and daily vegetable and fruit intake.

^c^ Model 3 further adjusted for body mass index, depression, stroke, and chronic disease comorbidity status.

Compared to participants with medium sleep duration, those sleeping > 8 hours per night showed poorer cognitive function (*β* = –1.13, *p* < 0.001), while those sleeping < 6 hours per night showed no significant difference in cognitive function (*β* = 0.30, *p* = 0.118). Expressed in age equivalents and compared to medium sleep duration, the effect of long sleep duration was equivalent to being 4 years older according to the composite cognition score (Table S6 in supporting information).

As shown in Figure 1, a significant non‐linear association between sleep duration and cognitive function also demonstrated that long sleep duration negatively affected cognitive function while short sleep duration did not (*p* value for non‐linearity < 0.001; the number of degrees of freedom was 5 in models based on GCV; Table S7 in supporting information).

We did not find an association between physical activity and cognitive function. Compared to participants reporting low physical activity, no significant difference in cognitive function was observed among participants reporting moderate physical activity (*β* = 0.34, *p *= 0.090) or high physical activity (*β* = 0.21, *p *= 0.129).

Figure 1 demonstrates a significant non‐linear association between physical activity and cognitive function (*p* value for non‐linearity = 0.024; the number of degrees of freedom was 8 in models based on GCV; Table S7), indicating that increased physical activity did not effectively improve cognitive function and even excessive physical activity could potentially impair cognitive function.

### 3.2 Stratified analysis by physical activity

Table 3 presents the results of the associations between sleep duration and cognitive function stratified by levels of physical activity in Model 3, with the results from Models 1 and 2 shown in Table S8 and Table S9 in supporting information, respectively. The findings across the three models were consistent. A significant interaction between sleep duration and physical activity for cognitive function was found (*p* value for interaction < 0.001). The detrimental associations between long sleep duration and cognitive function became significantly weaker among participants with higher levels of physical activity. The effect estimate of long sleep duration on cognitive function was –1.77 (*p* < 0.001), equivalent to 6.3 years of cognitive aging, among participants with low physical activity; –1.25 (*p* = 0.002), equivalent to 4.5 years of cognitive aging, among participants with moderate physical activity; and –0.54 (*p* = 0.008), equivalent to 1.9 years of cognitive aging, among participants with high physical activity. Additionally, the association between short sleep duration and cognitive function remained not significant among participants with moderate (*β* = ‐0.42, *p* = 0.437) or high (*β* = 0.00, *p* = 0.987) physical activity. However, among participants with low physical activity, participants who slept < 6 hours per night exhibited better cognitive function compared to participants with medium sleep duration (*β* = 0.85, *p* = 0.007).

**TABLE 3:** The adjusted associations of sleep duration and cognitive function at different levels of physical activity.

	Low physical activity		Moderate physical activity		High physical activity		
	*β*	*P*	*β*	*P*	*β*	*P*	*P* value for interaction
Sleep duration							
Short	0.85	0.007	−0.42	0.437	0.00	0.987	<0.001
Medium	Ref		Ref		Ref		
Long	−1.77	<0.001	−1.25	0.002	−0.54	0.008	

*Note*: Estimates from Model 3 adjusted for age, sex, education level, practice effect, rapid gait speed, sedentary behavior, sleep quality, composite cognitive score at concurrent wave, the interval between concurrent and next wave, marital status, employment status, socioeconomic status, smoking status, alcohol consumption, social cohesion, daily vegetable and fruit intake, body mass index, depression, stroke, and chronic disease comorbidity status.

### 3.3 Joint associations of sleep duration and physical activity with cognitive function

**FIGURE 2:**
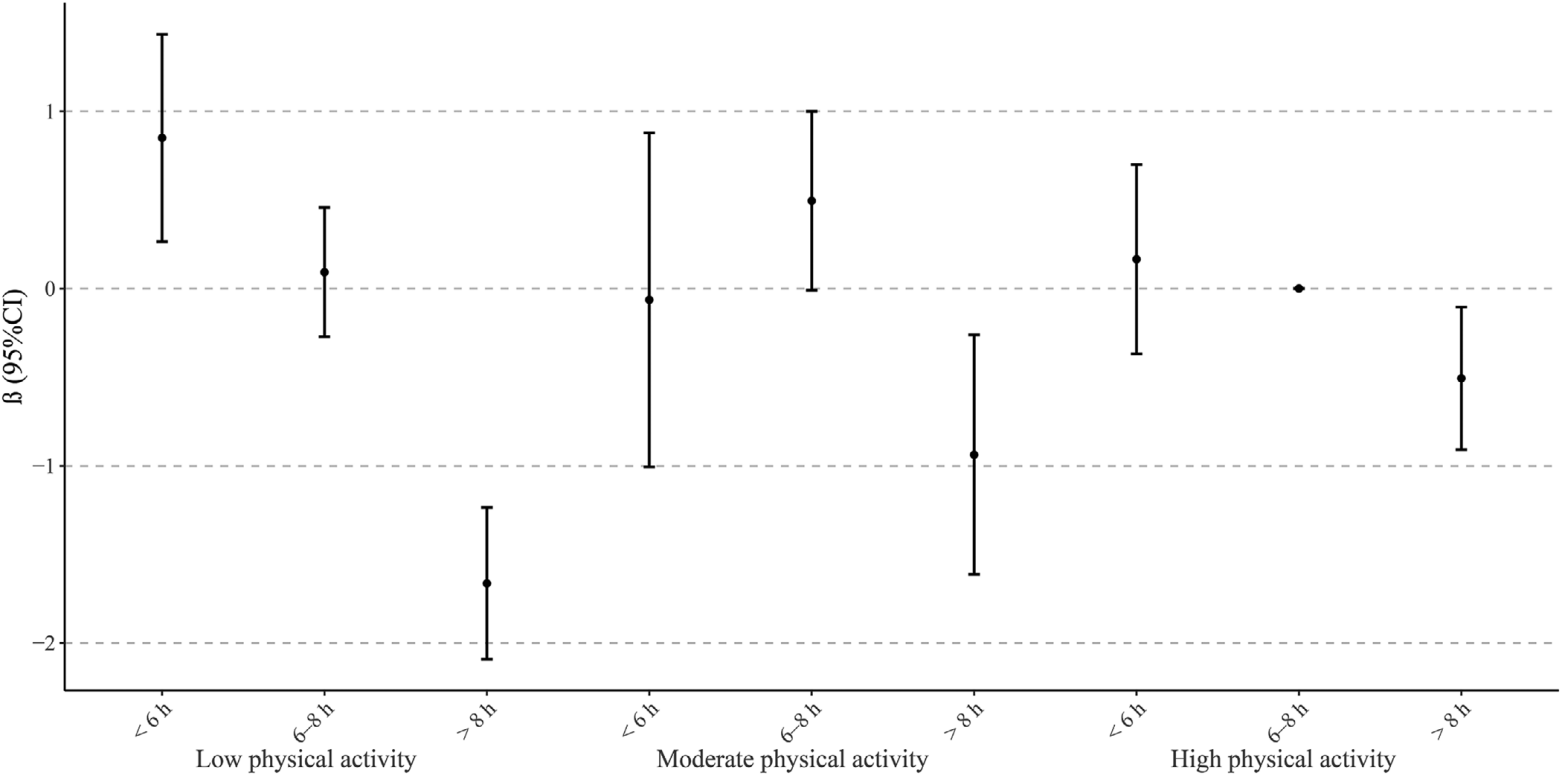
The adjusted associations between joint categories of sleep duration and physical activity with cognitive function. Estimates from Model 3. The reference group was participants who slept 6–8 hours per night and had high physical activity. CI, confidence interval

Table 4 shows the associations between joint categories of physical activity and sleep duration with cognitive function, with the results from Model 3 displayed in Figure 2. The pattern of associations was consistent across all three models but attenuated with the adjustment of additional confounders. Compared to participants with high physical activity and medium sleep duration, reporting either low physical activity and long sleep duration (*β* = –1.66, *p *< 0.001, age equivalents = 5.9 years), moderate activity and long sleep duration (*β* = –0.94, *p* = 0.007, age equivalents = 3.4 years), or high physical activity and long sleep duration (*β* = –0.51, *p* = 0.013, age equivalents = 1.8 years) was associated with poorer cognitive function. Relative to participants with high physical activity and medium sleep duration, reporting either moderate physical activity and medium sleep duration (*β* = 0.50, *p* = 0.5), low physical activity and medium sleep duration (*β* = 0.09, *p* = 0.621), high physical activity and short sleep duration (*β* = 0.17, *p* = 0.544), or moderate physical activity and short sleep duration (*β* = –0.06, *p* = 0.895) was not associated with cognitive function, but reporting low physical activity and short sleep duration was associated with better cognitive function (*β* = 0.85, *p* = 0.004).

**TABLE 4:** The adjusted associations between joint categories of sleep duration and physical activity with cognitive function.

		Model 1^a^		Model 2^b^		Model 3^c^	
		*β*	*P*	*β*	*P*	*β*	*P*
Low physical activity	Short sleep duration	0.96	0.002	0.82	0.006	0.85	0.004
	Medium sleep duration	0.29	0.127	0.10	0.610	0.09	0.621
	Long sleep duration	−1.54	<0.001	−1.71	<0.001	−1.66	<0.001
Moderate physical activity	Short sleep duration	0.01	0.981	−0.09	0.850	−0.06	0.895
	Medium sleep duration	0.82	0.002	0.49	0.055	0.50	0.055
	Long sleep duration	−0.81	0.020	−0.96	0.005	−0.94	0.007
High physical activity	Short sleep duration	0.18	0.504	0.13	0.637	0.17	0.544
	Medium sleep duration	Ref		Ref		Ref	
	Long sleep duration	−1.04	<0.001	−0.53	0.009	−0.51	0.013

^a^ Model 1 adjusted for age, sex, education level, practice effect, rapid gait speed, sedentary behavior, sleep quality, composite cognitive score at concurrent wave, and the interval between concurrent and next wave.

^b^ Model 2 additionally adjusted for marital status, employment status, socioeconomic status, smoking status, alcohol consumption, social cohesion, and daily vegetable and fruit intake.

^c^ Model 3 further adjusted for body mass index, depression, stroke, and chronic disease comorbidity status.

### 3.4 Sensitivity analysis

Similar findings were observed when analyzing the individual associations between sleep duration and physical activity at wave 1 with cognitive function at wave 3. The results showed a significant inverse association between long sleep duration and cognitive function, while the associations between short sleep duration and physical activity with cognitive function remained non‐significant (Table S10 in supporting information).

Results were substantively unchanged when excluding participants with MCI (Tables S11–S15 in supporting information), only including participants with complete data (Tables S16–S20) in supporting information, or using sleeping 7 to 8 hours per night as medium sleep duration and the reference group (Tables S21–S25 in supporting information).

## 4 DISCUSSION

The study, using longitudinal data from middle‐aged and older adults in China, had two key findings. First, long sleep duration was independently and prospectively associated with cognitive function, while short sleep duration and physical activity were not. Second, increased physical activity could lower the detrimental impact of long sleep duration on cognitive function.

The study found that participants who slept > 8 hours per night exhibited poorer cognition in the subsequent wave, equivalent to 4 years of cognitive aging, whereas no association was found for those who slept < 6 hours per night. This was verified in the curvilinear relationship between sleep duration and cognitive function and the sensitivity analysis with an average interval of 8.93 years between exposures and outcomes. The results were broadly similar to previous longitudinal studies,^31^, ^32^, ^33^, ^34^, ^35^ which suggested that long (rather than short) sleep duration was associated with worse cognition at follow‐up. For example, a study, using data from the Singapore Chinese Health Study, found that in cross‐sectional analysis, both long and short sleep duration were associated with cognitive impairment, whereas in longitudinal analysis, the association between long sleep duration and cognitive impairment remained significant with the exception for short sleep duration. And the study indicated that compared to the persistent recommended sleep duration (7 hours/day), persistent long sleep duration (≥ 9 hours/day) was significantly associated with cognitive impairment, while persistent short sleep duration (≤ 5 hours/day) was not.^34^ Additionally, a systematic review including 32 observational studies found that long (rather than short) sleep duration was linked to poorer cognition.^36^ However, some studies reported a significant association between short sleep duration and cognitive function. The reasons for this discrepancy across studies may be as follows. First, in the studies^8^, ^12^, ^33^ that reported adverse association between short sleep duration and cognitive function, the participants were more likely to be younger (mean age < 65 years) than other studies (mean age ≥ 65 years).^31^, ^32^, ^34^, ^35^, ^37^ In the present study, although the baseline mean age of participants was 62.1 years, the mean age of the follow‐up participants at wave 2 was 66.8 years. A study has indicated that as participants aged, the requirements for sleep duration decreased, leading to age‐specific “normal” sleep duration.^38^ Thus, a relatively short sleep duration (about 6 hours per night) might be normal for elderly people. Second, the discrepancy may be due partially to the adjustment of a wider range of confounders, especially sleep quality and depression. For example, a study among 10,768 Chinese adults revealed that when controlling for sleep quality, short sleep duration was no longer significantly associated with cognitive function, but long sleep duration was.^33^ This suggested that short sleep duration may be more indicative of poor sleep quality or sleep disturbances, as previously demonstrated.^39^ Furthermore, recent studies have shown that depression is linked to short sleep duration^40^ and cognitive decline.^41^ However, many studies failed to account for these factors. Third, the long preclinical phase of dementia may have led to reverse causation in studies with cross‐sectional design or short follow‐up period.^42^, ^43^ A study with biennial follow‐up surveys suggested that poorer cognitive function had a stronger impact on subsequent sleep duration than the reverse, using cross‐lagged models.^43^ Another study also indicated that short sleep duration may be a marker of cognitive impairment rather than a risk factor.^34^ Thus, compared to studies with a follow‐up period of > 5 years, studies with cross‐sectional design or a short follow‐up period were more likely to identify a statistically significant association between cognitive decline and short sleep duration.

Excessive sleep duration can lead to elevated neuroinflammation levels, including increased levels of proinflammatory factors (e.g., C‐reactive protein and interleukin 6), which is one of the crucial mechanisms for cognitive impairment.^44^, ^45^ In addition, long sleep duration may indicate sleep fragmentation, a significant contributor to brain aging.^46^, ^47^

Consistent with previous studies with a long follow‐up period,^7^, ^19^, ^48^ we found no evidence that higher levels of physical activity could improve cognitive function, which was confirmed in sensitivity analysis. These findings were supported by recent intervention studies^49^, ^50^, ^51^ that did not demonstrate a significant protective effect of increased physical activity on cognitive function. However, these conclusions contradicted numerous studies reporting significant associations between increased physical activity and better cognition. This inconsistency across studies may partially result from differences in study design.^9^, ^12^, ^52^, ^53^ The lengthy preclinical period of dementia may have led to alterations in physical activity.^4^, ^54^ Thus, studies with cross‐sectional design or short follow‐up period, typically < 10 years with dementia as outcome or < 5 years with cognitive decline before dementia as outcome, cannot completely avoid reverse causation.^54^ The significant protective effects of physical activity in studies with cross‐sectional design or short follow‐up times were possibly due to behavior changes caused by the preclinical period.^9^, ^52^, ^53^ For example, a study, including 431,924 participants from the UK Biobank study, suggested that the significant association between total physical activity and dementia was completely attenuated in a long follow‐up subgroup.^12^ In addition, some studies did not adjust for critical confounders, including health‐related behaviors and health conditions.^8^, ^19^, ^53^ For example, some studies incorporated stroke into chronic disease comorbidity status, which overlooked the stronger association and closer pathological mechanism link among physical activity, stroke, and cognitive function.^19^ Finally, different measurement methods for physical activity may also contribute to the mixed results. Measuring physical activity through self‐reporting could introduce measurement error, biasing the results.^55^ Using objective measurement of physical activity in future studies with a large‐scale nationally representative sample is warranted.

Interestingly, the non‐linear association between physical activity and cognitive function suggested that as physical activity increased, the composite cognitive score initially showed a quite slow increase, followed by a slightly faster decline, with a peak at approximately 4000 METs‐min per week. This trend aligned with previous findings that excessive moderate and vigorous physical activity in the elderly could generate a large amount of free radicals, leading to increased oxidative stress and further organ damage and pathological protein modifications in dementia.^12^, ^56^ This may play a crucial role in the inconsistency across studies. Further studies with large age‐span cohorts are warranted to explore the non‐linear effects of physical activity on cognitive function across strata of age and determine optimal levels of physical activity for different strata of age, especially for the elderly aged ≥ 65 years.

Increased physical activity appeared to alleviate the detrimental impacts of long sleep duration on cognitive function. The associations of joint categories of physical activity and sleep duration with cognitive function further quantified the combined effects of physical activity and sleep duration on cognitive function. Compared to participants with high physical activity and medium sleep duration, the effect size was gradually attenuated as the level of physical activity increased among participants with long sleep duration. These findings supported previous studies indicating that increased physical activity could reduce the increased risk of cognitive decline associated with excessive sleep duration.^8^, ^12^ Physical activity could improve sleep fragmentation^15^, ^57^ and reduce the elevated neuroinflammation level^58^ induced by excessive sleep duration.

The joint effects of physical activity and sleep duration underscored the importance of considering the current level of physical activity when developing potential strategies to improve sleep in older adults for the prevention of dementia.^59^ Especially for older adults who sleep > 8 hours per day, shortening sleep duration plus improving physical activity may be more effective.

Our analyses used a large‐scale nationally representative sample and adjusted for numerous confounders. Moreover, this study was conducted among participants without cognitive limitations and ensured a clear temporality from exposures to outcomes to minimize the possibility of reverse causation. However, this study had several limitations. First, the study relied on self‐reported sleep duration from only the two nights of sleep before the interview. Self‐reports on sleep duration may confuse time asleep with time in bed, leading to an overestimation of sleep duration. However, previous studies have shown that self‐reported sleep data could align well with objective measurements^33^, ^60^ and the discrepancy between self‐reported data and objective measures was smaller among Chinese individuals (49 minutes, 95% confidence interval: 37–61 minutes) compared to other ethnicities.^61^ Additionally, studies among SAGE participants provided reassurance that our measurement of sleep duration held crucial information for predicting health outcomes in the cohort.^40^, ^62^ Second, physical activity was assessed through self‐report rather than objective measures, potentially introducing measurement errors and misclassification of physical activity levels. Third, the effects found were about 1 point on a scale of 100, which was a relatively small effect. Fourth, despite our adjustment for a broader range of confounders, unmeasured covariates, such as apolipoprotein E status, sleep disorders, naps, and medication use affecting sleep (e.g., hypnotics, opioids, and psychotropic medications) might have introduced bias. Finally, the present study excluded participants who had died or did not complete the follow‐up survey, which may lead to selection bias. Therefore, our findings should be interpreted and generalized cautiously due to the inevitable limitations of an observational study.

## 5 CONCLUSION

In summary, this study highlighted the importance of sleep duration for cognitive function in middle‐aged and older adults. Long sleep duration was independently associated with poorer cognitive function in the future, while short sleep duration and physical activity were not. Furthermore, we also found that increased physical activity appeared to attenuate the detrimental effects of long sleep duration on cognitive function. As modifiable lifestyle factors, improving sleep duration and matching physical activity to sleep duration could be a promising intervention strategy for slowing cognitive decline among middle‐aged and older adults.

## CONFLICT OF INTEREST STATEMENT

The authors declare no conflicts of interest. Author disclosures are available in the supporting information.

## CONSENT STATEMENT

Informed consent was obtained from all participants before the interview.

## Supporting information

Supporting information

Supporting information
