# Supplementary material for: Individual and joint associations between sleep duration and physical activity with cognitive function: A longitudinal analysis among middle‐aged and older adults in China
Source: Alzheimers Dement. 2024 Dec 18;21(1):e14212. doi: 10.1002/alz.14212 (PMC11772731; doi:10.1002/alz.14212)
Supplement: Supplementary file 1 — Supporting information [file ALZ-21-e14212-s002.docx]

**
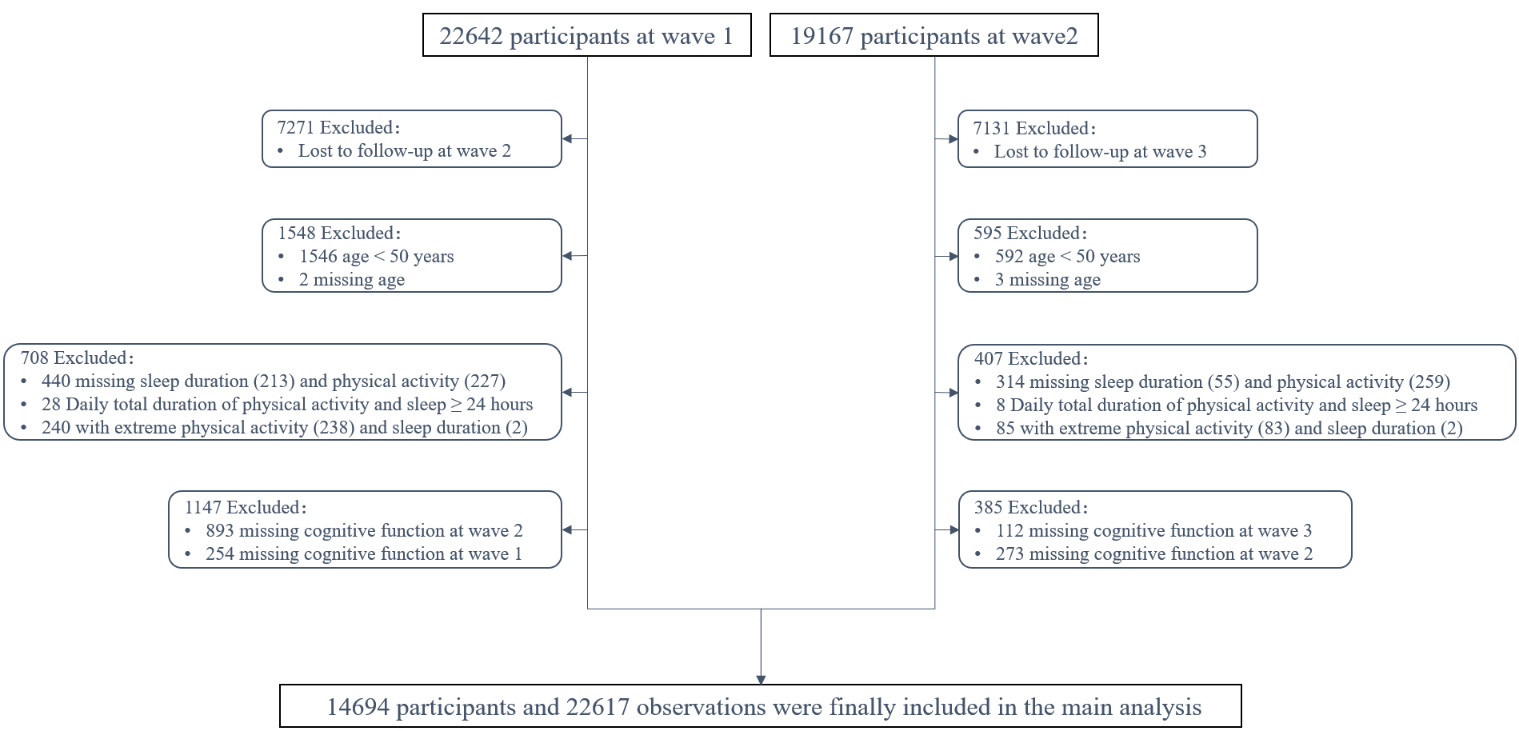
Supplementary** **material**

Figure S1 Flow chart of participants

Note: The study only selected participants completing individual questionnaire.

Table S1 The definition of covariates

| Covariates |  | Categories |  | Definition |
| --- | --- | --- | --- | --- |
| Education level |  | No formal education |  |  |
|  |  | Less than high school |  |  |
|  |  | High school or above |  |  |
| Marital status |  | Married |  | Currently married or cohabiting |
|  |  | Unmarried |  | Never married, separated, divorced, or widowed |
| Socioeconomic status |  | Low |  | Less than the median of the monthly household consumption spending per capital |
|  |  | High |  | More than or equal to the median of the monthly household consumption spending per capital |
| Alcohol consumption |  | Lifetime abstainers |  | Had never consumed a drink |
|  |  | No-heavy drinkers |  | Participants except lifetime abstainers and heavy drinkers |
|  |  | Heavy drinkers |  | Consuming 4 (female) or 5 (male) drinks on at least one day in the past week |
| Sedentary behavior ^a^ |  | Low |  | 0-2 hours of self-reported daily total sedentary time per day |
|  |  | Moderate |  | 2-4 hours of self-reported daily total sedentary time per day |
|  |  | High |  | More than 4 hours of self-reported daily total sedentary time per day |
| Daily vegetable and fruit intake |  | Insufficient |  | Less than 400 g per day |
|  |  | Sufficient |  | More than or equal to 400 g per day |
| Social cohesion index |  |  |  | The responses (response options ranging from “never” (coded as 1) to “daily” (coded as 5)) to nine questions regarding community engagement in the previous year (e.g., participation in religious services, clubs, societies, unions, etc.) were aggregated, with higher scores denoting greater social cohesion |
| Rapid gait speed, m/s |  |  |  | Assessed by timing the participant walking 4 meters at a rapid pace, as a proxy for physical function |
| Sleep quality |  |  |  | Participants were asked to rate their sleep quality on a scale of 1 to 5 (1 = very good quality, 5 = very poor quality) on each of the preceding two nights. The quality values from the two nights were averaged to calculate the typical sleep quality |
| BMI ^b^ |  | Underweight |  | <18.5 kg/m^2^ |
|  |  | Normal weight |  | 18.5 to 24.0 kg/m^2^ |
|  |  | Overweight and obesity |  | ≥24 kg/m^2^ |
| Depression ^1^ |  | No |  | Not meet the criteria of ‘yes’ |
|  |  | Yes |  | Self-reported a diagnosis of depression within the last 12 months OR past 12-month DSM-IV depression |
| Stroke ^2^ |  | No |  | Not meet the criteria of ‘yes’ |
|  |  | Yes |  | Self-reported a diagnosis of stroke within the last 12 months OR symptom-based diagnosis using algorithms |
| Chronic disease comorbidity status ^3^ |  | No |  | Hypertension was defined as meeting at least one of the following criteria: systolic blood pressure ≥140 mmHg, diastolic blood pressure ≥90 mmHg, or self-reported diagnosis. Diabetes was solely based on self-reported diagnosis. For angina, arthritis, asthma, chronic lung disease, and cataract, participants were considered to have the condition based on self-reported or symptom-based diagnosis using validated algorithms. The Rose questionnaire was used for angina, while other validated algorithms were used for arthritis, asthma, chronic lung disease, and cataract |
|  |  | 1-2 types |  |  |
|  |  | >=3 types |  |  |

Note: ^a^ Sedentary behavior was categorized based on self-reported daily total sedentary time; ^b^ BMI was calculated by dividing weight (in kilograms) by height (in meters) squared and categorized by the reference standard for the Chinese population.

Table S2 Missing rate of covariates

|  |  | Missing rate, % |
| --- | --- | --- |
|  |  |  |
| n=22617 | |  |
| Age | | 0 |
| Sex | | 0 |
| Education level | | 0 |
| Marital status | | 0 |
| Employment status | | 0 |
| Socioeconomic status | | 1 |
| Rapid gait speed | | 7 |
| sedentary behavior | | 0.4 |
| Sleep quality | | 0 |
| Smoking status | | 3.7 |
| Alcohol consumption | | 2.8 |
| Social cohesion index | | 0.5 |
| Daily vegetable and fruit intake | | 1.6 |
| BMI | | 5.5 |
| Depression | | 0.1 |
| Stroke | | 0.1 |
| Chronic disease comorbidity status | | 0 |
| Practice effect | | 0 |
| The interval | | 0 |
| Baseline composite cognitive score | | 0 |

Table S3 Baseline characteristics of participants across subgroups combining sleep duration and physical activity

|  |  |  | Total |  | Short sleep duration | | |  | Optimal sleep duration | | |  | Long sleep duration | | |
| --- | --- | --- | --- | --- | --- | --- | --- | --- | --- | --- | --- | --- | --- | --- | --- |
|  |  |  |  |  | Low PA | Moderate PA | High PA |  | Low PA | Moderate PA | High PA |  | Low PA | Moderate PA | High PA |
| n |  |  | 14694 |  | 846 | 287 | 1074 |  | 3000 | 1090 | 3860 |  | 1840 | 513 | 2184 |
| Sleep duration, hours per night, mean (SD) | | | 7.7 (1.4) |  | 5.4 (0.8) | 5.4 (0.8) | 5.4 (0.9) |  | 7.5 (0.6) | 7.5 (0.5) | 7.44 (0.5) |  | 9.2 (0.8) | 9.1 (0.8) | 9.2 (0.8) |
| PA, METs-min per week, mean (SD) | | | 2958.2 (4173.2) |  | 91.3 (170.4) | 879.3 (174.2) | 5457.7 (4484.6) |  | 101.9 (179.8) | 868.5 (180.8) | 5449.0 (4293.7) |  | 95.9 (173.4) | 865.8 (179.0) | 6579.8 (4731.6) |
| Age, years, mean (SD) | | | 62.1 (8.5) |  | 64.6 (9.2) | 63.4 (8.6) | 62.4 (8.2) |  | 61.9 (8.6) | 61.8 (8.4) | 60.7 (7.7) |  | 64.4 (9.5) | 62.9 (8.9) | 61.4 (8.0) |
| Sex, n (%) | | |  |  |  |  |  |  |  |  |  |  |  |  |  |
|  | Female | | 7980 (54.3) |  | 492 (58.2) | 166 (57.8) | 562 (52.3) |  | 1623 (54.1) | 583 (53.5) | 2106 (54.6) |  | 1008 (54.8) | 288 (56.1) | 1152 (52.7) |
|  | Male | | 6714 (45.7) |  | 354 (41.8) | 121 (42.2) | 512 (47.7) |  | 1377 (45.9) | 507 (46.5) | 1754 (45.4) |  | 832 (45.2) | 225 (43.9) | 1032 (47.3) |
| Education level | | |  |  |  |  |  |  |  |  |  |  |  |  |  |
|  | No former education | | 1856 (12.6) |  | 137 (16.2) | 44 (15.3) | 111 (10.3) |  | 304 (10.1) | 91 (8.3) | 379 (9.8) |  | 322 (17.5) | 70 (13.6) | 398 (18.2) |
|  | Less than high school | | 9334 (63.5) |  | 553 (65.4) | 161 (56.1) | 694 (64.6) |  | 1887 (62.9) | 625 (57.3) | 2451 (63.5) |  | 1181 (64.2) | 332 (64.7) | 1450 (66.4) |
|  | High school or above | | 3504 (23.8) |  | 156 (18.4) | 82 (28.6) | 269 (25.0) |  | 809 (27.0) | 374 (34.3) | 1030 (26.7) |  | 337 (18.3) | 111 (21.6) | 336 (15.4) |
| Marital status | | |  |  |  |  |  |  |  |  |  |  |  |  |  |
|  | Unmarried | | 1848 (12.6) |  | 158 (18.7) | 57 (19.9) | 154 (14.3) |  | 318 (10.6) | 128 (11.7) | 444 (11.5) |  | 274 (14.9) | 59 (11.5) | 256 (11.7) |
|  | Married | | 12846 (87.4) |  | 688 (81.3) | 230 (80.1) | 920 (85.7) |  | 2682 (89.4) | 962 (88.3) | 3416 (88.5) |  | 1566 (85.1) | 454 (88.5) | 1928 (88.3) |
| Employment status | | |  |  |  |  |  |  |  |  |  |  |  |  |  |
|  | Never worked | | 1623 (11.1) |  | 146 (17.3) | 33 (11.5) | 94 (8.8) |  | 473 (15.8) | 65 (6.0) | 256 (6.6) |  | 320 (17.4) | 36 (7.0) | 200 (9.2) |
|  | Retied | | 7134 (48.6) |  | 446 (52.7) | 160 (55.7) | 552 (51.4) |  | 1487 (49.6) | 624 (57.2) | 1898 (49.2) |  | 952 (51.8) | 283 (55.3) | 732 (33.6) |
|  | Working | | 5927 (40.4) |  | 254 (30.0) | 94 (32.8) | 428 (39.9) |  | 1038 (34.6) | 401 (36.8) | 1704 (44.2) |  | 567 (30.8) | 193 (37.7) | 1248 (57.2) |
| Socioeconomic status | | |  |  |  |  |  |  |  |  |  |  |  |  |  |
|  | Low | | 7183 (49.2) |  | 411 (49.2) | 138 (48.3) | 529 (49.5) |  | 1289 (43.3) | 384 (35.3) | 1866 (48.6) |  | 878 (48.2) | 222 (43.5) | 1466 (67.4) |
|  | High | | 7418 (50.8) |  | 424 (50.8) | 148 (51.7) | 539 (50.5) |  | 1686 (56.7) | 704 (64.7) | 1976 (51.4) |  | 945 (51.8) | 288 (56.5) | 708 (32.6) |
| Rapid gait speed, m/s | | | 1.4 (0.4) |  | 1.3 (0.4) | 1.4 (0.3) | 1.4 (0.3) |  | 1.4 (0.4) | 1.4 (0.4) | 1.4 (0.4) |  | 1.3 (0.4) | 1.4 (0.3) | 1.4 (0.3) |
| Sedentary behavior | | |  |  |  |  |  |  |  |  |  |  |  |  |  |
|  | Low | | 4496 (30.7) |  | 240 (28.5) | 68 (23.7) | 331 (30.9) |  | 911 (30.5) | 290 (26.6) | 1321 (34.3) |  | 555 (30.3) | 113 (22.1) | 667 (30.6) |
|  | Moderate | | 5558 (37.9) |  | 259 (30.8) | 92 (32.1) | 406 (37.9) |  | 988 (33.1) | 400 (36.7) | 1537 (39.9) |  | 695 (38.0) | 193 (37.8) | 988 (45.4) |
|  | High | | 4592 (31.4) |  | 343 (40.7) | 127 (44.3) | 334 (31.2) |  | 1089 (36.4) | 399 (36.6) | 993 (25.8) |  | 580 (31.7) | 205 (40.1) | 522 (24.0) |
| Sleep quality | | | 2.4 (0.7) |  | 3.0(0.8) | 3.0 (0.8) | 3.0 (0.9) |  | 2.4 (0.7) | 2.3 (0.6) | 2.3 (0.6) |  | 2.2 (0.7) | 2.2 (0.7) | 2.2 (0.7) |
| Smoking status | | |  |  |  |  |  |  |  |  |  |  |  |  |  |
|  | Never smokers | | 9645 (68.5) |  | 577 (70.3) | 195 (70.1) | 676 (65.4) |  | 2056 (73.2) | 719 (68.9) | 2475 (67.0) |  | 1265 (71.4) | 340 (69.7) | 1342 (62.9) |
|  | Former smokers | | 675 (4.8) |  | 47 (5.7) | 14 (5.0) | 64 (6.2) |  | 101 (3.6) | 34 (3.3) | 186 (5.0) |  | 85 (4.8) | 35 (7.2) | 109 (5.1) |
|  | Current smokers | | 3752 (26.7) |  | 197 (24.0) | 69 (24.8) | 293 (28.4) |  | 652 (23.2) | 290 (27.8) | 1033 (28.0) |  | 422 (23.8) | 113 (23.2) | 683 (32.0) |
| Alcohol consumption | | |  |  |  |  |  |  |  |  |  |  |  |  |  |
|  | Lifetime abstainers | | 10024 (71.3) |  | 618 (75.3) | 199 (71.1) | 671 (64.6) |  | 2165 (77.2) | 768 (74.1) | 2488 (67.4) |  | 1389 (78.4) | 372 (76.4) | 1354 (63.4) |
|  | Non-heavy drinkers | | 3301 (23.5) |  | 158 (19.2) | 68 (24.3) | 302 (29.1) |  | 528 (18.8) | 232 (22.4) | 1021 (27.7) |  | 304 (17.2) | 91 (18.7) | 597 (27.9) |
|  | Heavy drinkers | | 741 (5.3) |  | 45 (5.5) | 13 (4.6) | 65 (6.3) |  | 111 (4.0) | 37 (3.6) | 183 (5.0) |  | 78 (4.4) | 24 (4.9) | 185 (8.7) |
| Social cohesion index | | | 14.8 (3.7) |  | 14.8 (4.3) | 14.0 (3.0) | 15.1 (3.9) |  | 14.2 (3.6) | 14.6 (3.4) | 15.4 (3.7) |  | 14.1 (3.5) | 14.3 (3.4) | 15.1 (3.4) |
| Daily vegetable and fruit intake | | |  |  |  |  |  |  |  |  |  |  |  |  |  |
|  | Insufficient | | 2916 (20.3) |  | 175 (21.6) | 61 (21.4) | 202 (19.0) |  | 702 (24.4) | 206 (19.2) | 612 (16.1) |  | 462 (26.1) | 107 (21.2) | 389 (18.0) |
|  | Sufficient | | 11436 (79.7) |  | 635 (78.4) | 224 (78.6) | 862 (81.0) |  | 2170 (75.6) | 869 (80.8) | 3200 (83.9) |  | 1311 (73.9) | 398 (78.8) | 1767 (82.0) |
| BMI, kg/m2 | | |  |  |  |  |  |  |  |  |  |  |  |  |  |
|  | Underweight (<18.5) | | 445 (3.2) |  | 39 (4.8) | 11 (3.9) | 49 (4.7) |  | 56 (2.0) | 31 (3.0) | 109 (2.9) |  | 58 (3.4) | 11 (2.2) | 81 (3.8) |
|  | Normal (18.5-24) | | 6613 (46.9) |  | 358 (43.7) | 131 (46.5) | 546 (51.9) |  | 1261 (44.9) | 472 (45.0) | 1806 (48.2) |  | 774 (44.9) | 223 (45.5) | 1042 (49.1) |
|  | Overweight and obesity (>=24) | | 7033 (49.9) |  | 423 (51.6) | 140 (49.6) | 457 (43.4) |  | 1489 (53.1) | 546 (52.0) | 1834 (48.9) |  | 890 (51.7) | 256 (52.2) | 998 (47.1) |
| Depression | | |  |  |  |  |  |  |  |  |  |  |  |  |  |
|  | No | | 14532 (99.0) |  | 824 (97.7) | 282 (98.3) | 1051 (97.9) |  | 2967 (99.1) | 1084 (99.4) | 3829 (99.3) |  | 1822 (99.3) | 512 (99.8) | 2161 (99.1) |
|  | Yes | | 141 (1.0) |  | 19 (2.3) | 5 (1.7) | 22 (2.1) |  | 28 (0.9) | 6 (0.6) | 27 (0.7) |  | 13 (0.7) | 1 (0.2) | 20 (0.9) |
| Stroke | |  |  |  |  |  |  |  |  |  |  |  |  |  |  |
|  | No | | 14193 (96.7) |  | 801 (94.9) | 275 (95.8) | 1033 (96.2) |  | 2885 (96.4) | 1060 (97.2) | 3755 (97.4) |  | 1766 (96.1) | 493 (96.1) | 2125 (97.4) |
|  | Yes | | 482 (3.3) |  | 43 (5.1) | 12 (4.2) | 41 (3.8) |  | 107 (3.6) | 30 (2.8) | 101 (2.6) |  | 72 (3.9) | 20 (3.9) | 56 (2.6) |
| Chronic disease comorbidity status | | |  |  |  |  |  |  |  |  |  |  |  |  |  |
|  | No | | 4063 (27.7) |  | 179 (21.2) | 59 (20.6) | 289 (26.9) |  | 910 (30.3) | 306 (28.1) | 1152 (29.8) |  | 455 (24.7) | 134 (26.1) | 579 (26.5) |
|  | 1-2 types | | 9193 (62.6) |  | 516 (61.0) | 178 (62.0) | 630 (58.7) |  | 1814 (60.5) | 689 (63.2) | 2394 (62.0) |  | 1208 (65.7) | 323 (63.0) | 1441 (66.0) |
|  | >=3 types | | 1438 (9.8) |  | 151 (17.8) | 50 (17.4) | 155 (14.4) |  | 276 (9.2) | 95 (8.7) | 314 (8.1) |  | 177 (9.6) | 56 (10.9) | 164 (7.5) |
| Baseline composite cognitive score | | | 61.8 (10.2) |  | 58.5 (10.9) | 60.7 (11.3) | 62.6 (10.3) |  | 61.7 (10.1) | 63.8 (10.2) | 63.5 (9.8) |  | 59.6 (10.4) | 62.3 (10.8) | 61.0 (9.7) |

Abbreviations: BMI, body mass index; SD, standard deviation; PA, physical activity.


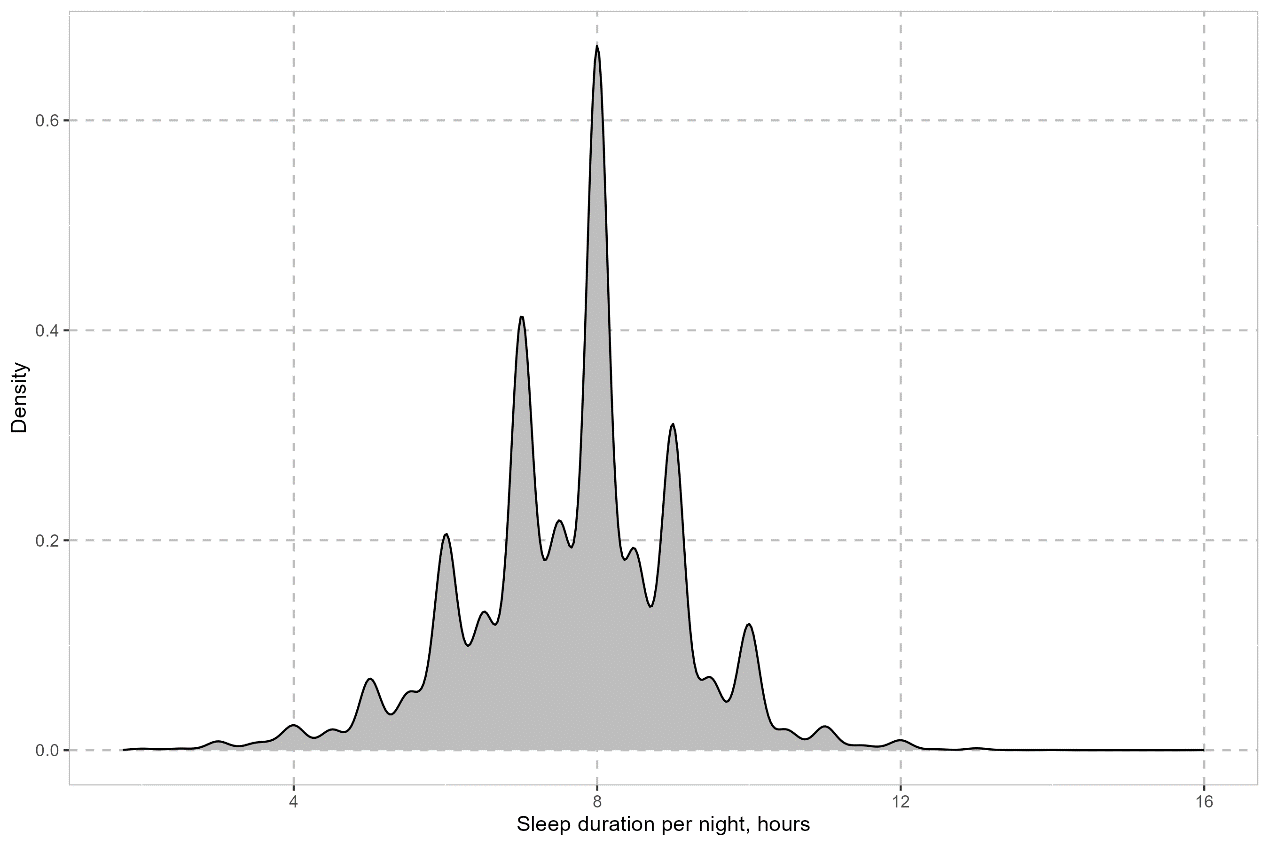


Figure S2 The distribution of sleep duration per night


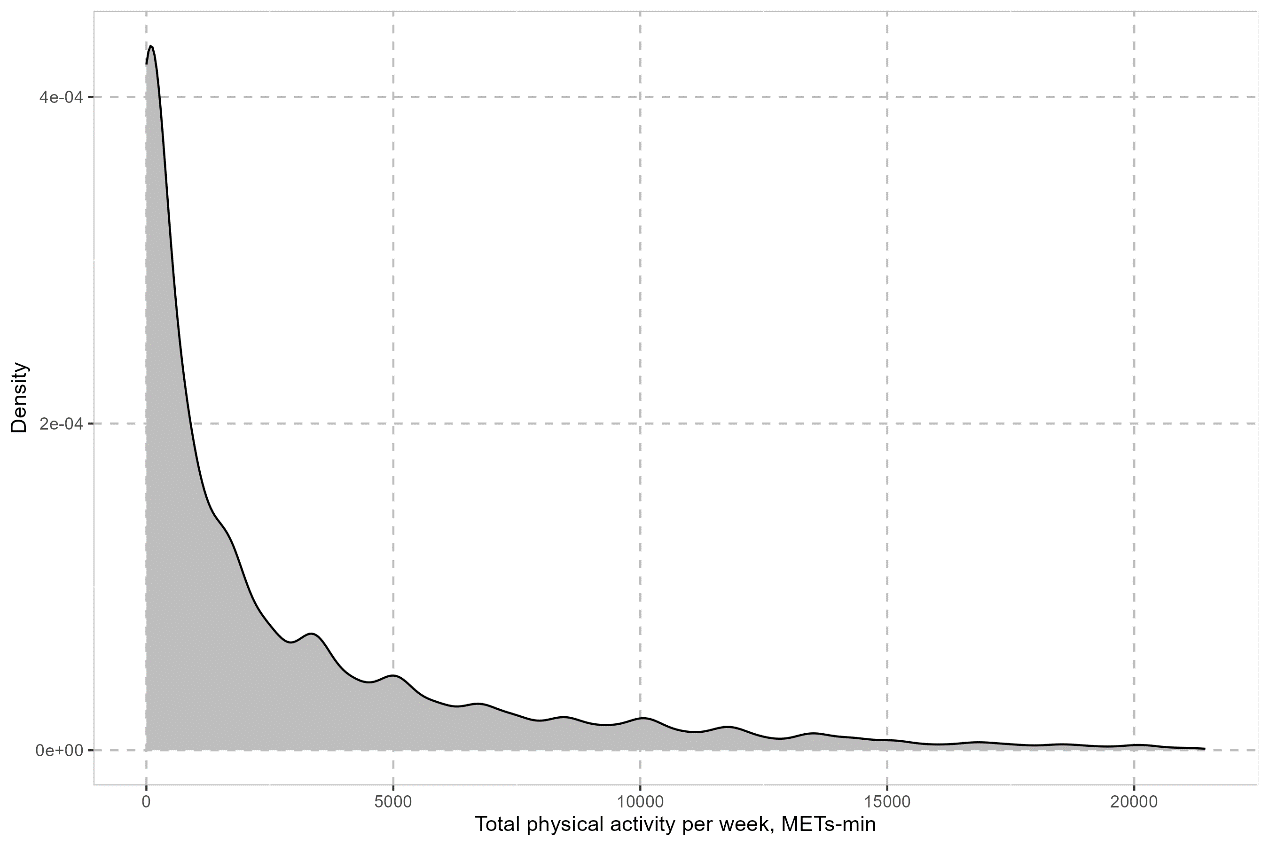


Figure S3 The distribution of physical activity per week

Table S4 The correlations between sleep duration, physical activity, and sedentary behavior

|  |  | Sleep duration | |  | Physical activity | |  | Sedentary behavior | |
| --- | --- | --- | --- | --- | --- | --- | --- | --- | --- |
|  |  | r | *p* |  | r | *p* |  | r | *p* |
| Sleep duration |  | 1.000 |  |  |  |  |  |  |  |
| Physical activity |  | -0.002 | 0.761 |  | 1.000 |  |  |  |  |
| Sedentary behavior |  | -0.013 | 0.059 |  | -0.091 | < 0.001 |  | 1.000 |  |

Abbreviations: r, correlation coefficients

Table S5 Generalized variance-inflation factor (GVIF) and GVIF^(1/(2*Df)) of all variables

|  |  | GVIF | Df | GVIF^(1/(2*Df)) |
| --- | --- | --- | --- | --- |
| Sleep duration |  | 1.168 | 2 | 1.040 |
| Physical activity |  | 1.120 | 2 | 1.029 |
| Age |  | 1.712 | 1 | 1.308 |
| Sex |  | 2.221 | 1 | 1.490 |
| Education level |  | 1.514 | 2 | 1.109 |
| Practice effect |  | 1.709 | 1 | 1.307 |
| Rapid gait speed |  | 1.231 | 1 | 1.110 |
| Sedentary behavior |  | 1.067 | 2 | 1.016 |
| Sleep quality |  | 1.183 | 1 | 1.088 |
| Composite cognitive score at concurrent wave |  | 1.403 | 1 | 1.185 |
| The interval between concurrent and next wave |  | 1.678 | 1 | 1.296 |
| Marital status |  | 1.134 | 1 | 1.065 |
| Employment status |  | 1.488 | 2 | 1.104 |
| Socioeconomic status |  | 1.300 | 1 | 1.140 |
| Smoking status |  | 2.134 | 2 | 1.209 |
| Alcohol consumption |  | 1.535 | 2 | 1.113 |
| Social cohesion index |  | 1.076 | 1 | 1.037 |
| Daily vegetable and fruit intake |  | 1.265 | 1 | 1.125 |
| BMI |  | 1.066 | 2 | 1.016 |
| Depression |  | 1.022 | 1 | 1.011 |
| Stroke |  | 1.033 | 1 | 1.016 |
| Chronic disease comorbidity status |  | 1.184 | 2 | 1.043 |

Abbreviations: GVIF, generalized variance-inflation factor; Df, degree of freedom.

To assess the robustness of the results, we examined the multicollinearity among covariates in the mixed models conducted in the present study. Generalized variance-inflation factor (GVIF) and GVIF^(1/(2*Df)) were calculated using R (version 4.2.3) with the “car” package. GVIF^(1/(2*Df)) of all covariates were less than 2 (Table x), equal to an ordinary variance-inflation factor (VIF) < 4 for one-coefficient variables, which suggested no evidence of multicollinearity in the present study.

Table S6 The associations of age with cognitive function

|  |  | Model 1^a^ | |  | Model 2^b^ | |  | Model 3^c^ | |
| --- | --- | --- | --- | --- | --- | --- | --- | --- | --- |
|  |  | β | *p* |  | β | *p* |  | β | *p* |
| Age, years | |  |  |  |  |  |  |  |  |
|  |  | -0.24 | < 0.001 |  | -0.29 | < 0.001 |  | -0.28 | < 0.001 |

Table S7 The results from generalized cross-validation for the degrees of freedom

|  |  | Physical activity | | |  | Sleep duration | | |
| --- | --- | --- | --- | --- | --- | --- | --- | --- |
|  |  | RMSE | R-squared | MAE |  | RMSE | R-squared | MAE |
| Degrees of freedom | |  |  |  |  |  |  |  |
|  | 4 | 9.321066 | 0.285366 | 7.350974 |  | 9.321483 | 0.285302 | 7.35122 |
|  | **5** | 9.321079 | 0.2853639 | 7.350991 |  | **9.321147** | **0.285354** | **7.351153** |
|  | 6 | 9.321113 | 0.2853587 | 7.350991 |  | 9.321287 | 0.2853321 | 7.351192 |
|  | 7 | 9.320991 | 0.2853774 | 7.350921 |  | 9.32123 | 0.2853407 | 7.351155 |
|  | **8** | **9.320891** | **0.285393** | **7.350853** |  | 9.321263 | 0.2853357 | 7.351189 |
|  | 9 | 9.320917 | 0.2853888 | 7.350872 |  | 9.321259 | 0.2853364 | 7.351172 |
|  | 10 | 9.320942 | 0.2853849 | 7.350893 |  | 9.321219 | 0.2853424 | 7.351153 |
|  | 11 | 9.320896 | 0.285392 | 7.35086 |  | 9.321249 | 0.2853379 | 7.351153 |

Table S8 The adjusted associations of sleep duration and cognitive function at different levels of physical activity in model 1

|  |  | |  | | Low physical activity | | | |  | | Moderate physical activity | | | |  | | High physical activity | | | |  | | *P* value for interaction | |
| --- | --- | --- | --- | --- | --- | --- | --- | --- | --- | --- | --- | --- | --- | --- | --- | --- | --- | --- | --- | --- | --- | --- | --- | --- |
|  |  | |  | | β | | *p* | |  | | β | | *p* | |  | | β | | *p* | |  | |  |  |
| Sleep duration | |  | |  | |  | |  | |  | |  | |  | |  | |  | |  | |  | |  |
|  | Short | |  | | 0.82 | | 0.010 | |  | | -0.68 | | 0.216 | |  | | 0.02 | | 0.953 | |  | | 0.004 | |
|  | Medium | |  | | Ref | | | |  | | Ref | | | |  | | Ref | | | |  | |  |  |
|  | Long | |  | | -1.80 | | < 0.001 | |  | | -1.50 | | < 0.001 | |  | | -1.06 | | 0.001 | |  | |  |  |

Table S9 The adjusted associations of sleep duration and cognitive function at different levels of physical activity in model 2

|  |  |  | Low physical activity | |  | Moderate physical activity | |  | High physical activity | |  | *P* value for interaction |
| --- | --- | --- | --- | --- | --- | --- | --- | --- | --- | --- | --- | --- |
|  |  |  | β | *p* |  | β | *p* |  | Β | *p* |  |  |
| Sleep duration | |  |  |  |  |  |  |  |  |  |  |  |
|  | Short |  | 0.82 | 0.009 |  | -0.47 | 0.382 |  | -0.04 | 0.889 |  | < 0.001 |
|  | Medium |  | Ref | |  | Ref | |  | Ref | |  |  |
|  | Long |  | -1.81 | < 0.001 |  | -1.25 | 0.002 |  | -0.57 | 0.005 |  |  |

The first sensitivity analysis: The individual associations between sleep duration and physical activity at wave 1 and composite cognitive score at wave 3

Table S10 The individual associations of sleep duration and physical activity at wave 1 with cognitive function at wave 3

|  |  | Model 1 | |  | Model 2 | |  | Model 3 | |
| --- | --- | --- | --- | --- | --- | --- | --- | --- | --- |
|  |  | β | *p* |  | β | *p* |  | β | *p* |
| Physical activity | |  |  |  |  |  |  |  |  |
|  | Low | Ref | |  | Ref | |  | Ref | |
|  | Moderate | 0.35 | 0.302 |  | 0.21 | 0.540 |  | 0.21 | 0.539 |
|  | High | 0.35 | 0.139 |  | 0.44 | 0.070 |  | 0.45 | 0.062 |
| Sleep duration | |  |  |  |  |  |  |  |  |
|  | Short | -0.36 | 0.246 |  | -0.35 | 0.266 |  | -0.34 | 0.273 |
|  | Medium | Ref | |  | Ref | |  | Ref | |
|  | Long | -0.96 | < 0.001 |  | -0.61 | 0.011 |  | -0.59 | 0.013 |

The second sensitivity analysis: The analyses where participants with mild cognitive impairment in at least two waves were identified and excluded

Table S11 The individual associations of sleep duration and physical activity with cognitive function among participants without mild cognitive impairment

|  |  | Model 1 | |  | Model 2 | |  | Model 3 | |
| --- | --- | --- | --- | --- | --- | --- | --- | --- | --- |
|  |  | β | *p* |  | β | *p* |  | β | *p* |
| Physical activity | |  |  |  |  |  |  |  |  |
|  | Low | Ref | |  | Ref | |  | Ref | |
|  | Moderate | 0.25 | 0.237 |  | 0.29 | 0.180 |  | 0.28 | 0.197 |
|  | High | -0.24 | 0.096 |  | 0.10 | 0.525 |  | 0.09 | 0.564 |
| Sleep duration | |  |  |  |  |  |  |  |  |
|  | Short | 0.27 | 0.184 |  | 0.28 | 0.183 |  | 0.31 | 0.141 |
|  | Medium | Ref | |  | Ref | |  | Ref | |
|  | Long | -1.49 | < 0.001 |  | -1.20 | < 0.001 |  | -1.17 | < 0.001 |

Table S12 The adjusted associations of sleep duration and cognitive function at different levels of physical activity in model 3 among participants without mild cognitive impairment

|  |  |  | Low physical activity | |  | Moderate physical activity | |  | High physical activity | |  | *P* value for  interaction |
| --- | --- | --- | --- | --- | --- | --- | --- | --- | --- | --- | --- | --- |
|  |  |  | β | *p* |  | β | *p* |  | β | *p* |  |  |
| Sleep duration | |  |  |  |  |  |  |  |  |  |  |  |
|  | Short |  | 0.77 | 0.027 |  | -0.52 | 0.362 |  | 0.10 | 0.740 |  | < 0.001 |
|  | Medium |  | Ref | |  | Ref | |  | Ref | |  |  |
|  | Long |  | -1.83 | < 0.001 |  | -1.40 | 0.002 |  | -0.59 | 0.006 |  |  |

Table S13 The adjusted associations of sleep duration and cognitive function at different levels of physical activity in model 2 among participants without mild cognitive impairment

|  |  |  | Low physical activity | |  | Moderate physical activity | |  | High physical activity | |  | *P* value for interaction |
| --- | --- | --- | --- | --- | --- | --- | --- | --- | --- | --- | --- | --- |
|  |  |  | β | *p* |  | β | *p* |  | β | *p* |  |  |
| Sleep duration | |  |  |  |  |  |  |  |  |  |  |  |
|  | Short |  | 0.76 | 0.030 |  | -0.57 | 0.320 |  | 0.05 | 0.864 |  | < 0.001 |
|  | Medium |  | Ref | |  | Ref | |  | Ref | |  |  |
|  | Long |  | -1.88 | < 0.001 |  | -1.39 | 0.002 |  | -0.62 | 0.004 |  |  |

Table S14 The adjusted associations of sleep duration and cognitive function at different levels of physical activity in model 1 among participants without mild cognitive impairment

|  |  |  | Low physical activity | |  | Moderate physical activity | |  | High physical activity | |  | *P* value for interaction |
| --- | --- | --- | --- | --- | --- | --- | --- | --- | --- | --- | --- | --- |
|  |  |  | β | *p* |  | β | *p* |  | β | *p* |  |  |
| Sleep duration | |  |  |  |  |  |  |  |  |  |  |  |
|  | Short |  | 0.73 | 0.028 |  | -0.70 | 0.218 |  | 0.12 | 0.673 |  | 0.017 |
|  | Medium |  | Ref | |  | Ref | |  | Ref | |  |  |
|  | Long |  | -1.83 | < 0.001 |  | -1.72 | < 0.001 |  | -1.13 | < 0.001 |  |  |

Table S15 The adjusted associations between joint categories of sleep duration and physical activity with cognitive function among participants without mild cognitive impairment

|  |  | Model 1 | |  | Model 2 | |  | Model 3 | |
| --- | --- | --- | --- | --- | --- | --- | --- | --- | --- |
|  |  | β | *p* |  | β | *p* |  | β | *p* |
| Low physical  activity | Short sleep duration | 1.03 | 0.001 |  | 0.93 | 0.004 |  | 0.96 | 0.003 |
|  | Medium sleep duration | 0.42 | 0.033 |  | 0.24 | 0.245 |  | 0.24 | 0.232 |
|  | Long sleep duration | -1.41 | < 0.001 |  | -1.61 | < 0.001 |  | -1.56 | < 0.001 |
| Moderate physical  activity | Short sleep duration | 0.02 | 0.961 |  | -0.14 | 0.790 |  | -0.10 | 0.838 |
|  | Medium sleep duration | 0.84 | 0.002 |  | 0.60 | 0.028 |  | 0.60 | 0.028 |
|  | Long sleep duration | -0.95 | 0.008 |  | -0.92 | 0.012 |  | -0.91 | 0.014 |
| High physical  activity | Short sleep duration | 0.29 | 0.304 |  | 0.23 | 0.417 |  | 0.27 | 0.350 |
|  | Medium sleep duration | Ref | |  | Ref | |  | Ref | |
|  | Long sleep duration | -1.13 | < 0.001 |  | -0.60 | 0.006 |  | -0.57 | 0.008 |

The third sensitivity analysis: The analyses among participants with complete covariate data

Table S16 The individual associations of sleep duration and physical activity with cognitive function among participants with complete data

|  |  | Model 1 | |  | Model 2 | |  | Model 3 | |
| --- | --- | --- | --- | --- | --- | --- | --- | --- | --- |
|  |  | β | *p* |  | β | *p* |  | β | *p* |
| Physical activity | |  |  |  |  |  |  |  |  |
|  | Low | Ref | |  | Ref | |  | Ref | |
|  | Moderate | 0.30 | 0.158 |  | 0.33 | 0.118 |  | 0.32 | 0.132 |
|  | High | -0.20 | 0.168 |  | 0.14 | 0.344 |  | 0.13 | 0.378 |
| Sleep duration | |  |  |  |  |  |  |  |  |
|  | Short | 0.20 | 0.317 |  | 0.19 | 0.362 |  | 0.22 | 0.291 |
|  | Medium | Ref | |  | Ref | |  | Ref | |
|  | Long | -1.47 | < 0.001 |  | -1.19 | < 0.001 |  | -1.15 | < 0.001 |

Table S17 The adjusted associations of sleep duration and cognitive function at different levels of physical activity in model 3 among participants with complete data

|  |  |  | Low physical activity | |  | Moderate physical activity | |  | High physical activity | |  | *P* value for  interaction |
| --- | --- | --- | --- | --- | --- | --- | --- | --- | --- | --- | --- | --- |
|  |  |  | β | *p* |  | β | *p* |  | β | *p* |  |  |
| Sleep duration | |  |  |  |  |  |  |  |  |  |  |  |
|  | Short |  | 0.79 | 0.025 |  | -0.67 | 0.240 |  | -0.03 | 0.915 |  | < 0.001 |
|  | Medium |  | Ref | |  | Ref | |  | Ref | |  |  |
|  | Long |  | -1.81 | < 0.001 |  | -1.31 | 0.003 |  | -0.59 | 0.006 |  |  |

Table S18 The adjusted associations of sleep duration and cognitive function at different levels of physical activity in model 2 among participants with complete data

|  |  |  | Low physical activity | |  | Moderate physical activity | |  | High physical activity | |  | *P* value for interaction |
| --- | --- | --- | --- | --- | --- | --- | --- | --- | --- | --- | --- | --- |
|  |  |  | β | *p* |  | β | *p* |  | β | *p* |  |  |
| Sleep duration | |  |  |  |  |  |  |  |  |  |  |  |
|  | Short |  | 0.77 | 0.027 |  | -0.73 | 0.204 |  | -0.07 | 0.802 |  | < 0.001 |
|  | Medium |  | Ref | |  | Ref | |  | Ref | |  |  |
|  | Long |  | -1.86 | < 0.001 |  | -1.30 | 0.003 |  | -0.62 | 0.004 |  |  |

Table S19 The adjusted associations of sleep duration and cognitive function at different levels of physical activity in model 1 among participants with complete data

|  |  |  | Low physical activity | |  | Moderate physical activity | |  | High physical activity | |  | *P* value for interaction |
| --- | --- | --- | --- | --- | --- | --- | --- | --- | --- | --- | --- | --- |
|  |  |  | β | *p* |  | β | *p* |  | β | *p* |  |  |
| Sleep duration | |  |  |  |  |  |  |  |  |  |  |  |
|  | Short |  | 0.77 | 0.022 |  | -0.85 | 0.136 |  | 0.02 | 0.950 |  | 0.011 |
|  | Medium |  | Ref | |  | Ref | |  | Ref | |  |  |
|  | Long |  | -1.81 | < 0.001 |  | -1.61 | < 0.001 |  | -1.13 | < 0.001 |  |  |

Table S20 The adjusted associations between joint categories of sleep duration and physical activity with cognitive function among participants with complete data

|  |  | Model 1 | |  | Model 2 | |  | Model 3 | |
| --- | --- | --- | --- | --- | --- | --- | --- | --- | --- |
|  |  | β | *p* |  | β | *p* |  | β | *p* |
| Low physical  activity | Short sleep duration | 0.98 | 0.002 |  | 0.85 | 0.009 |  | 0.88 | 0.007 |
|  | Medium sleep duration | 0.35 | 0.074 |  | 0.17 | 0.416 |  | 0.17 | 0.400 |
|  | Long sleep duration | -1.45 | < 0.001 |  | -1.66 | < 0.001 |  | -1.61 | < 0.001 |
| Moderate physical  activity | Short sleep duration | -0.11 | 0.821 |  | -0.29 | 0.562 |  | -0.26 | 0.609 |
|  | Medium sleep duration | 0.83 | 0.002 |  | 0.58 | 0.034 |  | 0.59 | 0.033 |
|  | Long sleep duration | -0.88 | 0.016 |  | -0.86 | 0.020 |  | -0.85 | 0.022 |
| High physical  activity | Short sleep duration | 0.18 | 0.530 |  | 0.10 | 0.720 |  | 0.14 | 0.632 |
|  | Medium sleep duration | Ref | |  | Ref | |  | Ref | |
|  | Long sleep duration | -1.13 | < 0.001 |  | -0.59 | 0.006 |  | -0.57 | 0.009 |

The fourth sensitivity analysis: The analyses using sleeping 7-8 hours per night as medium sleep duration

Table 21 The individual associations of sleep duration and physical activity with cognitive function using sleeping 7-8 hours per night as medium sleep duration

|  |  | Model 1^a^ | |  | Model 2^b^ | |  | Model 3^c^ | |
| --- | --- | --- | --- | --- | --- | --- | --- | --- | --- |
|  |  | β | *p* |  | β | *p* |  | β | *p* |
| Physical activity | |  |  |  |  |  |  |  |  |
|  | Low | Ref | |  | Ref | |  | Ref | |
|  | Moderate | 0.40 | 0.050 |  | 0.31 | 0.120 |  | 0.31 | 0.114 |
|  | High | -0.11 | 0.434 |  | 0.15 | 0.289 |  | 0.16 | 0.249 |
| Sleep duration | |  |  |  |  |  |  |  |  |
|  | Short | 0.30 | 0.077 |  | 0.36 | 0.032 |  | 0.38 | 0.022 |
|  | Medium | Ref | |  | Ref | |  | Ref | |
|  | Long | -1.39 | <0.001 |  | -1.10 | <0.001 |  | -1.06 | <0.001 |

Table 22 The adjusted associations of sleep duration and cognitive function at different levels of physical activity in model 3 using sleeping 7-8 hours per night as medium sleep duration

|  |  |  | Low physical activity | |  | Moderate physical activity | |  | High physical activity | |  | *P* value for  interaction |
| --- | --- | --- | --- | --- | --- | --- | --- | --- | --- | --- | --- | --- |
|  |  |  | β | *p* |  | β | *p* |  | β | *p* |  |  |
| Sleep duration | |  |  |  |  |  |  |  |  |  |  |  |
|  | Short |  | 0.49 | 0.069 |  | 0.03 | 0.946 |  | 0.33 | 0.171 |  | <0.001 |
|  | Optimal |  | Ref | |  | Ref | |  | Ref | |  |  |
|  | Long |  | -1.74 | <0.001 |  | -1.15 | 0.005 |  | -0.48 | 0.023 |  |  |

Table 23 The adjusted associations of sleep duration and cognitive function at different levels of physical activity in model 2 using sleeping 7-8 hours per night as medium sleep duration

|  |  |  | Low physical activity | |  | Moderate physical activity | |  | High physical activity | |  | *P* value for interaction |
| --- | --- | --- | --- | --- | --- | --- | --- | --- | --- | --- | --- | --- |
|  |  |  | β | *p* |  | β | *p* |  | β | *p* |  |  |
| Sleep duration | |  |  |  |  |  |  |  |  |  |  |  |
|  | Short |  | 0.4673 | 0.086 |  | 0.0031 | 0.995 |  | 0.3312 | 0.163 |  | <0.001 |
|  | Optimal |  | Ref | |  | Ref | |  | Ref | |  |  |
|  | Long |  | -1.7896 | <0.001 |  | -1.1524 | 0.005 |  | -0.4839 | 0.021 |  |  |

Table 24 The adjusted associations of sleep duration and cognitive function at different levels of physical activity in model 1 using sleeping 7-8 hours per night as medium sleep duration

|  |  |  | Low physical activity | |  | Moderate physical activity | |  | High physical activity | |  | *P* value for interaction |
| --- | --- | --- | --- | --- | --- | --- | --- | --- | --- | --- | --- | --- |
|  |  |  | β | *p* |  | β | *p* |  | β | *p* |  |  |
| Sleep duration | |  |  |  |  |  |  |  |  |  |  |  |
|  | Short |  | 0.40 | 0.144 |  | -0.19 | 0.682 |  | 0.33 | 0.169 |  | 0.025 |
|  | Optimal |  | Ref | |  | Ref | |  | Ref | |  |  |
|  | Long |  | -1.84 | <0.001 |  | -1.45 | 0.001 |  | -0.97 | <0.001 |  |  |

Table 25 The adjusted associations between joint categories of sleep duration and physical activity with cognitive function using sleeping 7-8 hours per night as medium sleep duration

|  |  | Model 1^a^ | |  | Model 2^b^ | |  | Model 3^c^ | |
| --- | --- | --- | --- | --- | --- | --- | --- | --- | --- |
|  |  | β | *p* |  | β | *p* |  | β | *p* |
| Low physical  activity | Short sleep duration | 0.76 | 0.004 |  | 0.71 | 0.006 |  | 0.72 | 0.005 |
|  | Medium sleep duration | 0.42 | 0.037 |  | 0.29 | 0.151 |  | 0.25 | 0.221 |
|  | Long sleep duration | -1.44 | <0.001 |  | -1.52 | <0.001 |  | -1.49 | <0.001 |
| Moderate physical  activity | Short sleep duration | 0.56 | 0.151 |  | 0.46 | 0.232 |  | 0.47 | 0.229 |
|  | Medium sleep duration | 0.86 | 0.002 |  | 0.56 | 0.044 |  | 0.55 | 0.051 |
|  | Long sleep duration | -0.71 | 0.044 |  | -0.80 | 0.021 |  | -0.78 | 0.025 |
| High physical  activity | Short sleep duration | 0.44 | 0.067 |  | 0.44 | 0.065 |  | 0.45 | 0.059 |
|  | Medium sleep duration | Ref | |  | Ref | |  | Ref | |
|  | Long sleep duration | -0.94 | <0.001 |  | -0.43 | 0.042 |  | -0.41 | 0.053 |

**References:**

1. Kessler RC, Ustün TB. The World Mental Health (WMH) Survey Initiative Version of the World Health Organization (WHO) Composite International Diagnostic Interview (CIDI). *Int J Methods Psychiatr Res*. 2004;13(2):93-121. doi:10.1002/mpr.168

2. Garin N, Koyanagi A, Chatterji S, et al. Global Multimorbidity Patterns: A Cross-Sectional, Population-Based, Multi-Country Study. *J Gerontol A Biol Sci Med Sci*. Feb 2016;71(2):205-14. doi:10.1093/gerona/glv128

3. Sun Y, Shi L, Bao Y, Sun Y, Shi J, Lu L. The bidirectional relationship between sleep duration and depression in community-dwelling middle-aged and elderly individuals: evidence from a longitudinal study. *Sleep Med*. Dec 2018;52:221-229. doi:10.1016/j.sleep.2018.03.011
